# Exploring Cardiovascular Involvement in Tuberous Sclerosis: Insights for Pediatric Clinicians

**DOI:** 10.3390/children11060674

**Published:** 2024-06-02

**Authors:** Cecilia Lazea, Ioana Țaranu, Sorana D. Bolboacă

**Affiliations:** 1 Pediatric Clinic 1, Emergency Pediatric Hospital, Calea Moților, No. 68, 400370 Cluj-Napoca, Romania; cecilialazea@umfcluj.ro; 2 Department Mother and Child, Iuliu Hațieganu University of Medicine and Pharmacy, Calea Moților, No. 68, 400370 Cluj-Napoca, Romania; 3 Department of Medical Informatics and Biostatistics, Iuliu Hațieganu University of Medicine and Pharmacy, Louis Pasteur Str., No. 6, 400349 Cluj-Napoca, Romania; sbolboaca@umfcluj.ro

**Keywords:** tuberous sclerosis, cardiac rhabdomyomas, arrhythmias, screening, follow-up

## Abstract

Tuberous sclerosis is a rare genetic disorder involving mainly the nervous and cardiovascular systems. The early recognition of the cardiovascular manifestations by the pediatrician allows an appropriate management and therefore enhances the quality of life of the affected children. Cardiac rhabdomyomas and the associated arrhythmias are the first cardiac features and they might represent a diagnosis challenge given their wide spectrum of clinical manifestations. We aimed to provide the paediatric practitioners with current knowledge regarding the cardiovascular complications in children with tuberous sclerosis. We overviewed the antenatal and postnatal evolution of cardiovascular manifestations, the systematic screening and long-term follow-up strategy of cardiac rhabdomyomas and arrhythmias in children with tuberous sclerosis.

## 1. Introduction

Tuberous sclerosis (TS) is a rare disorder resulting from de novo mutations within the TSC1 or TSC2 gene or following an autosomal dominant pattern of inheritance [1]. The estimated prevalence in Europe ranges from 1 to 25,000 to 1 in 11,300 newborns, while in the United States of America 1 in 6000 newborns is affected by this disorder [2]. Tuberous sclerosis is classically characterized by neurological manifestations (i.e., cognitive disability and epilepsy), cutaneous features (i.e., fibromas, hypomelanotic macules and connective tissue nevi) and hamartomatous lesions of various organ systems [2].

The latest paediatric guideline of the International Tuberous Sclerosis Complex Consensus Group published in 2021 revised the criteria previously provided in 2013 for definite or possible diagnosis [3]. A definite diagnosis of TS requires two major features or one major feature and ≥2 minor features, whereas a possible diagnosis emerges from the presence of one major feature or ≥2 minor features. The latter definition also comprises the association of ≥2 angiomyolipomas and lymphangiomyomatosis (major criteria) in the absence of other criteria. Notably, the genetic diagnosis might be considered in the presence of pathogenic sequence variants in TSC1 or TSC2 genes that prevent the protein production or inactivate the protein function [1].

TSC1 and TSC2 mutations are independent criteria sufficient to establish the diagnosis in spite of a large disease variability from mild to severe forms of disease [1,3,4]. However, Tyburczy et al. [5] showed that 10 to 15% of patients with clinical manifestations of TS might have no mutation identified by conventional molecular testing. Mosaicism and intronic mutations were identified in most of these patients [5]. Hamartin and tuberin, the tumour-suppressor proteins encoded by TSC1 and TSC2 genes, form a heterodimeric complex which upregulates the activity of the mammalian target of rapamycin complex 1, a critical regulator of cell growth, also known as mTORC1 [6]. In the affected tumour cells, the activation of the mTORC1 signaling network results in increased cell growth and nucleotide synthesis with decreased autophagy [6].

TuberOus SClerosis registry to increase disease Awareness (TOSCA) was the largest worldwide analysis of TS manifestations and interventions, including a cohort of 2093 patients with TS from over 31 countries and spanning over 5 years (2012–2017) [7]. More than half (57%) of the included patients were diagnosed in a neuropediatric or pediatric clinic, these data highlighting the critical role of the pediatrician regarding the early diagnosis and thus, the prevention of severe manifestations of TS [7].

The timing of clinical presentation in TS is of particular interest for clinicians since an early diagnosis might significantly reduce the severity of neurological degradation by enabling earlier surveillance and treatment [8]. Thus, timely treatment is of high importance given the deleterious influence of the TS diagnosis and management challenges on the quality of life of the affected children and their caregivers [9,10].

Cardiac rhabdomyoma, one of the major clinical criteria of TS, is the earliest manifestation of TS, commonly reported within the first year of life (mean age of 0.8 ± 1.4 years) [11]. In a prospective longitudinal analysis enrolling 130 infants, cardiac rhabdomyoma was the initial presenting feature of TS, out passing the neurological and cutaneous findings prevalence [12]. Similarly, across the age groups represented in TOSCA, cardiac tumours were reported together with hypomelanotic macules and central nervous system tumours starting from age under 2 years, while the diagnosis of TSC in adulthood was based on the presence of renal angiomyolipomas, facial angiofibromas, fibrous cephalic plaques and shagreen patches [7]. In addition to the above evidence, Pearsson et al. revealed a scarcity of standardized management and follow-up of the cardiac, renal and ophthalmological manifestations of TS patients [13]. These arguments represent the main incentives for our present review.

The purpose of our narrative review was to provide the paediatrician with current knowledge regarding the cardiovascular manifestations in children with tuberous sclerosis. We overviewed the diagnosis of tuberous sclerosis in paediatric patients, and highlighted the natural evolution, screening, and management of cardiovascular manifestations in children with tuberous sclerosis.

## 2. Cardiac Manifestations in Tuberous Sclerosis

Cardiovascular and neurological manifestations in TS remain the major concerns of the paediatrician treating children affected by TS. Rhabdomyoma, the wide spectrum of arrhythmia, and vascular abnormalities are the most common cardiovascular manifestations in TS. Thus, systematic screening and long-term follow-up is mandatory.

### 2.1. Definition and Manifestations of Cardiac Rhabdomyomas in Children with Tuberous Sclerosis

Initially described by von Recklinghausen in 1862, cardiac rhabdomyomas are benign hamartomatous growths [14]. These circumscribed tumours are characterized by the presence of pathognomonic spider cells with a central granular cytoplasm with myofibrils and elongated projections to the periphery. Rhabdomyoma cells are glycogen-rich atypical cardiomyocytes, different from Purkinje fibres or myocardial working cells and, although distended by glycogen deposits, they distinguish from cardiac myocytes in glycogen storage disease [14]. These benign hamartomas are the most prevalent primary cardiac tumours in childhood, accounting for 53.9% of the general prevalence of primary cardiac tumours in a recent meta-analysis investigating their management, followed by fibroma and myxoma [15].

#### 2.1.1. Manifestations during Foetal Period

Cardiac rhabdomyoma, the first manifestation of TS in the foetal period, appears at 20 to 30 weeks of gestation and the number and size might increase during gestation due to maternal hormonal changes. More specifically, in utero exposure to an elevated oestrogen serum level might lead to the activation of mTORC1 and mTORC2 signaling network that further increases the tumour growth. The tumour can regress during gestation or postnatally by apoptosis [16,17,18]. Although the majority are clinically silent, depending on the tumour’s location in the ventricular septum, they might manifest as various types of arrhythmias, outflow obstruction (for tumours located in the upper interventricular septum), heart failure and pericardial effusion (for giant cardiac rhabdomyomas), or foetal hydrops secondary to heart failure [17,18,19,20].

The rate of detection of cardiac rhabdomyomas by ultrasound cardiac screening in the second and third trimester is increased. Table 1 shows the detection rate of cardiac rhabdomyomas in fetuses with regard to the number of tumours and the previously established diagnosis of TS.

The differential diagnosis for foetal primary cardiac tumours includes benign such as fibromas, myxomas and hemangiomas or malignant tumours, which are rare [17]. Several studies have demonstrated that multiple rhabdomyomas are a strong predictor for clinically diagnosed TS in neonatal and early infancy, underlying the importance of foetal ultrasound screening for a precocious diagnosis and potential early intervention [17,21,22,23].

#### 2.1.2. Manifestations during Postnatal Period

Postnatally, cardiac rhabdomyomas might present a large spectrum of clinical and electrophysiological changes in close relation with the intracardiac location of the tumour. Clinical findings may include heart murmur, arrhythmia, heart failure, cyanosis, syncope, hemodynamic instability, altered mental status or cardiac arrest with sudden death [16,20,24].

Echocardiography is the primary imaging tool for assessing the cardiac involvement in TS. Rhabdomyomas typically manifest as multiple nodular, homogenous, hyperechoic masses that can be detected in the ventricles (without predilection for a particular one), but they may also be located in the atria. They can protrude into the cardiac chambers leading to outflow tract or valvular obstruction or can compress the coronary arteries resulting in myocardial ischemia. Rarely, in case of multiple tumours, ventricular function might be also affected. Their dimensions are variable, from a few millimeters to several centimeters. Over 90% of patients with TS have multiple such tumours, but solitary tumours have also been described [25,26]. In case of a solitary tumour, careful echocardiographic examination is imposed in order to detect other small masses [21].

#### 2.1.3. Evolution of Cardiac Rhabdomyoma

The progression of cardiac rhabdomyomas follows a complex age-dependent pattern in patients with TS with an incidence that ranges from 100% in neonates and during the first months of life, to 35% in children over 10 years old and a mean age at diagnosis of 3.1 years [7,23,27]. Slow disappearance of these tumours is determined by vacuolar degeneration and apoptosis of the cytoplasm [21,28].

Spontaneous regression marks the evolution of the cardiac rhabdomyoma during childhood in the first two years of life, the rate of complete regression being variable, between 31% and 80% [17,29,30,31,32]. Jozwiak et al. reported that the tumours regressed in approximately 70% of 55 paediatric cases, when “watchful waiting” was pursued during a median follow-up period of 3.5 years, ranging from 0.5 to 18 years [33].

**Figure 1:**
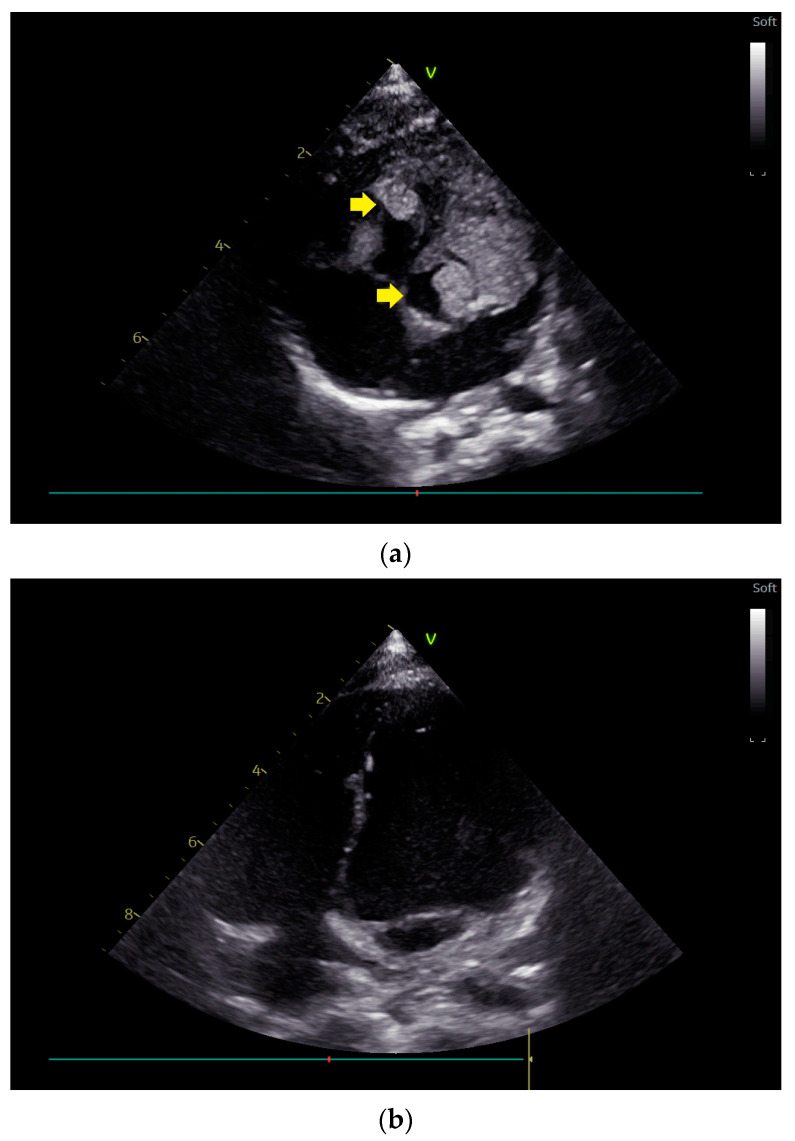
Transthoracic echocardiography showing multiple ventricular rhabdomyomas in the left and right ventricle (yellow arrows) in a neonate diagnosed with tuberous sclerosis (**a**) and their spontaneous partial regression at the age of 2 years (**b**).

Figure 1 presents echocardiographic images of multiple rhabdomyoma in a neonate diagnosed with TS, in the left and right ventricle (a) with a spontaneous partial regression in the first two years of life (b).

Few clinical studies have reported inconstant recurrence of the tumours during adolescence related to hormonal changes and extremely rare development of a new cardiac rhabdomyoma beyond infancy, suggesting the need for routine echocardiographic surveillance in patients with apparently stable or regressing cardiac rhabdomyomas [33,34]. In more than half of adults with TS myocardial fatty foci have been revealed by computed tomography, while ultrasound erroneously identified these lesions as rhabdomyomas [32,35]. In adults, the most common echocardiographic abnormalities described are multiple focal areas of increased intramyocardial echogenicity, valvular and myocardial function abnormalities [36].

Au et al. have found that missense mutations in the TSC2 gene in 325 patients with TS have a higher risk for more severe phenotypes of tuberous sclerosis, including a higher frequency of cardiac rhabdomyomas than the protein truncation mutation in the TSC1 gene [37].

The reported incidence of cardiac rhabdomyoma in children with TS range from 42% to 100%, while the reported regression ranges from 31% to 80% (Table 2).

### 2.2. Arrhythmia in Patients with Tuberous Sclerosis

#### 2.2.1. Clinical Manifestations

Cardiac rhabdomyomas might constitute substrates for arrhythmias in children in close relation with their location, number, and dimension. Table 3 presents the spectrum of electrocardiographic modifications in postnatally diagnosed cardiac rhabdomyomas based on paediatric case reports and case series of TS.

In children with tuberous sclerosis, pre-excitation, supraventricular tachyarrhythmia and ventricular ectopic beats were the most often reported rhythm abnormalities, followed by prolonged PR interval and atrioventricular block [45].

Clinical manifestations include palpitations, fatigue, syncope (which can be mistaken for “drop attacks” and seizures), cardiac arrest and sudden death. Of note, arrhythmia can represent a life-threatening manifestation during neonatal period.

**Figure 2:**
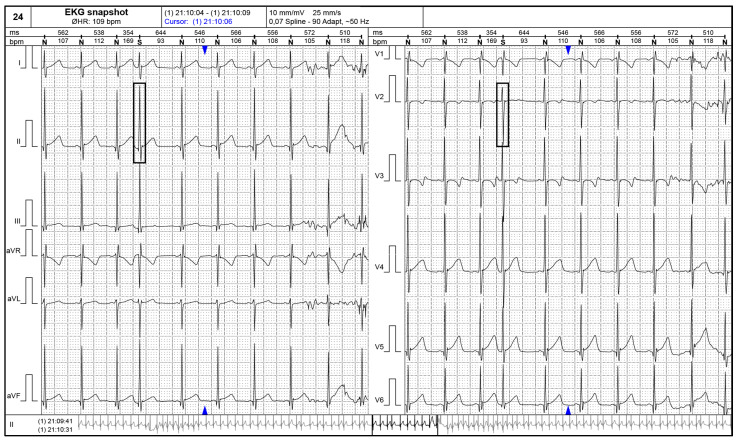
24–h ECG Holter monitoring showing a supraventricular ectopic beat (black rectangular shapes) in a neonate with tuberous sclerosis.

**Figure 3:**
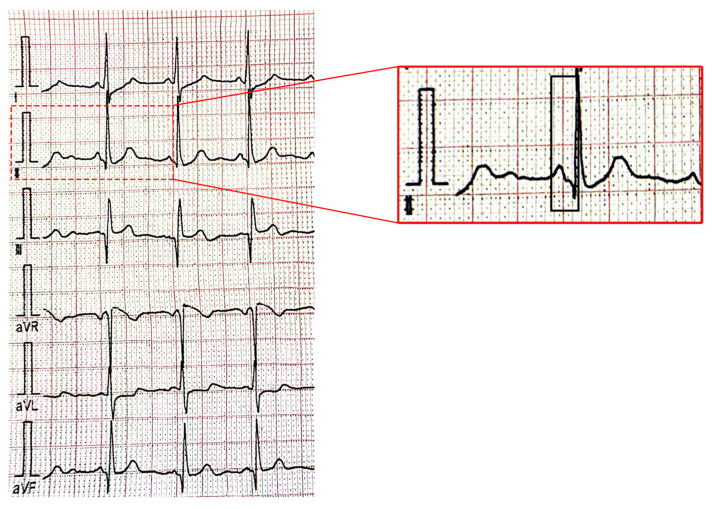
Electrocardiogram showing a short PR interval (80 milliseconds) (black rectangular shape) in a 2–year–old patient with tuberous sclerosis: first figure showing peripheral derivations of the ECG leads and second figure showing the magnified DII peripheral lead.

Figure 2 and Figure 3 present supraventricular ectopic beats in a neonate with TS and electrocardiographic changes (short PR interval) in a 2–years–old patient with TS.

#### 2.2.2. Incidence of Arrhythmia in Children with Tuberous Sclerosis

Arrhythmia has been reported variably, depending on the age of the patient and the type of arrhythmia, from 30–50% in infants to 5.6% in children and adolescents [21,32] (Table 4).

#### 2.2.3. Pathophysiological Substrates of Arrhythmia

The mechanism of arrhythmia is generally linked to the location of the cardiac rhabdomyoma [48]. For instance, dysfunction of the sinus and atrioventricular node is related to the mechanism of bradycardia, while accessory atrioventricular connection and ventricular tachycardia are involved in tachycardias [48].

##### Preexcitation Syndromes and Paroxysmal Supraventricular Tachycardias

Electrocardiographic evidences for multiple accessory atrioventricular connections has been documented in multiple cardiac rhabdomyomas [53]. Intramural rhabdomyomas may interrupt the conduction pathways leading to ectopic electrical foci or an accessory electrical circuit [33].

The pathophysiological mechanism for preexcitation and paroxysmal supra-ventricular tachycardias implies the similarity of the hamartomatous cells to normal Purkinje cells in rhabdomyomas located at the atrioventricular junction, where they function as accessory pathways by bypassing the atrioventricular node [41,54]. These accessory pathways can subsequently disappear or become inactive when the underlying rhabdomyoma decreases in size (spontaneously or after mTOR inhibitors therapy). However, in rare instances, when the involution of the tumour interrupts the atrioventricular node bundle of HIS junction, these arrhythmias might evolve toward a third degree atrioventricular block [47].

##### Arrhythmia Related to Ventricular Obstruction

Ventricular hypertrophy depends on the size of tumour and location and is usually present in the context of giant tumours located in the ventricular cavities. Benyounes et al. reported a case of a 10-year-old girl with two cardiac rhabdomyomas inserted on the right side of the interventricular septum, resulting in pressure overload of the ventricle without affecting the cardiac output [55].

Direct compression of coronary arteries or myocardial injury can explain repolarization abnormalities as dome-shaped ST-segment elevation by direct pressure of rhabdomyoma [51].

The significant size of the rhabdomyoma and its communication with the interventricular septum and ventricular outflow tract can also explain Brugada phenocopy [52].

## 3. Vascular Manifestations in Tuberous Sclerosis

Concerning the vascular involvement in TS, patients may develop aneurysmal dilatations of the vessels, large and medium size arterial stenotic-occlusive disease and dysplasia of the small vessels. More specifically, the incidence of aneurysms is twice in TS patients than in general population and they can involve aorta, carotid, axillary, renal, iliac, femoral and pulmonary arteries [56,57,58,59,60,61,62,63]. Although pulmonary artery aneurysms are very rare, doctors should suspect them in TS patients with dyspnea or chest pain [64,65]. Taking into consideration that the aneurysms associated TS are usually large at the presentation, they could be detected in the first year of life or later. Still, because of rapid progression and high risk of rupture, they can represent a major and fatal complication if unrecognized [58,59]. Therefore, healthcare providers should routinely check for this complication in patients with TS and promptly repair it [60].

Dysplastic and degenerative changes within the arterial wall, increased proliferation of smooth muscle cells, and a disorganized structure of the elastic layer contribute to the aneurysmal dilatation in TS [66]. Abnormality of connective tissue such as loss of elastin fibers has been suggested as a possible cause of these changes [67].

## 4. Screening for Cardiac Manifestations in Tuberous Sclerosis

The International Tuberous Sclerosis Consensus Group recommended a close surveillance for cardiac manifestations in newborns and children under 3 years old newly diagnosed with TS or suspected. In fetuses diagnosed with cardiac rhabdomyomas, the foetal ultrasound should be followed by foetal echocardiography in the third trimester in order to establish the risk of heart failure at birth. At least one echocardiogram is recommended for these newborns to rule out any possible hemodynamic impairment [1,45].

Similarly, infants and children under 3 years old diagnosed with TS should be screened for rhabdomyomas and arrhythmias via at least a 12- to 15-lead electrocardiogram and echocardiography. Finally, a baseline ECG is recommended in all ages due to the risk of underlying conduction defects [1,45].

Regarding cardiac long-term surveillance, asymptomatic paediatric patients should be evaluated every 1 to 3 years by an echocardiogram until regression of cardiac rhabdomyomas, whereas a more frequent assessment might be necessary for symptomatic patients. For asymptomatic patients of all ages, it is recommended to perform an ECG every 3 to 5 years to monitor for conduction defects or to perform a 24- to 48-h Holter monitoring in symptomatic patients [1,45].

Ultrasound is sufficient for both screening and definitive assessment of abdominal aneurysm, but computed tomography or magnetic resonance imaging should be used for vascular assessment in the case of lesions outside abdominal aorta.

**Figure 4:**
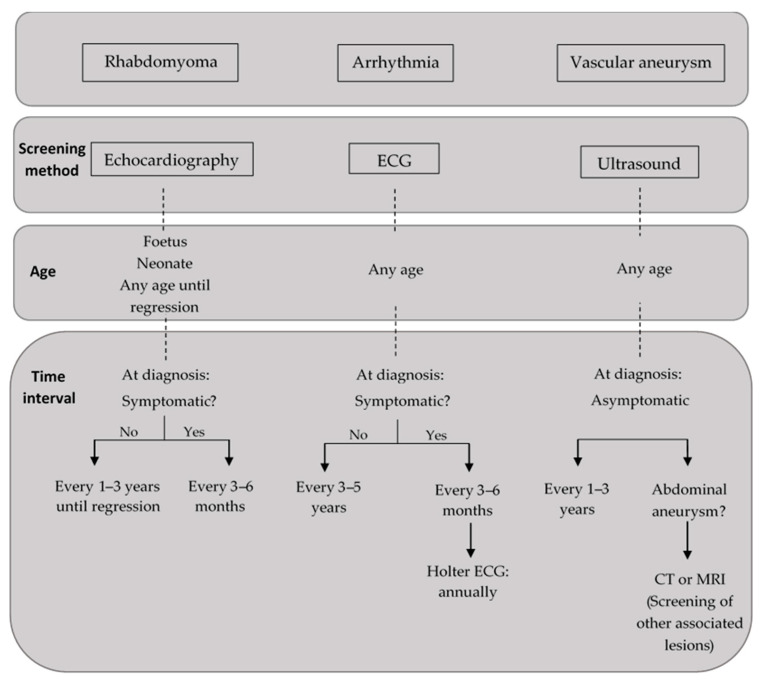
Screening and surveillance of the cardiovascular manifestations in patients with tuberous sclerosis (diagram constructed based on data previously reported [1,45]; ECG: electrocardiogram, CT: computed tomography, MRI: medical resonance imaging).

Figure 4 presents the recommendations of International Tuberous Sclerosis Consensus Group [1] for cardiovascular lesions screening and surveillance in patients with TS.

## 5. Treatment According to the Evolution of Cardiovascular Manifestations

Surgical intervention (complete or partial cardiac rhabdomyoma resection) is indicated in rare circumstances, in whom heart failure installs as a complication of inflow or outflow obstruction [34,45,68]. Nevertheless, neonates and infants with heart failure are critically ill, so the risk of surgical intervention is not at all negligible. Moreover, in some cases, rhabdomyomas may be inoperable due to their location, size, and relation with valves or coronary arteries. Benyounes et al. estimated that 3.72% (12/322) children with TS had undergone cardiac rhabdomyoma surgery [55].

A multicenter analysis conducted by the European Congenital Heart Surgeons Association in 2012 revealed that 71.8% of children who underwent a surgical treatment for rhabdomyoma had congestive heart failure, arrhythmias or neurological symptoms following embolization [69].

mTOR inhibitors (sirolimus and everolimus) are mammalian target of rapamycin inhibitors that can inhibit growth-driven cell proliferation. A significant reduction in seizure frequency was found in children with TS with treatment-refractory seizures after receiving everolimus as adjuvant therapy [70,71,72]. Concerning the cardiovascular manifestations, mTOR therapy has also been evaluated in children with refractory heart failure and subsequent hemodynamic instability [73,74,75,76,77,78]. However, in the absence of comparative clinical trials, selection of a specific mTOR inhibitor had followed the published evidence to date for the specific disease manifestation [79]. Patients with large and symptomatic inoperable rhabdomyoma may be candidates for the mTOR inhibitor therapy. Oral sirolimus initiated after 27 weeks of gestation for progressively obstructive foetal multiple cardiac rhabdomyomas improved the ventricular function, whilst a complete tumour regression was obtained in the first two years of treatment [18,80,81]. A multicenter study of 217 patients had demonstrated that sirolimus was responsible for the disappearance of cardiac rhabdomyoma within an average period of 9 months of medication in 60% of children, with higher tumour disappearance rates in younger age group (children under 4 years of age) [82]. Still, the optimal duration of mTOR treatment is still unknown. Studies have proven the lack of standardization of the dose and duration of treatment of mTOR inhibitors used in children and infants with rhabdomyomas [83,84,85]. Discontinuation of everolimus therapy in an infant with multiple rhabdomyomas and refractory and recurrent episodes of supraventricular tachycardia resulted in tumour size rebound and recurrence of arrhythmic episodes [86].

ORACLE trial is the first clinical trial assessing the efficacy of everolimus therapy in children with symptomatic cardiac rhabdomyoma. According to this study, inclusion criteria are symptomatic rhabdomyomas such as severe ventricular or supraventricular arrhythmia, moderate to severe obstruction of the outflow tracts (pulmonary or aortic), heart failure with preserved or reduced ejection fraction, pericardial effusion, hemodynamically and neurologically stable patient [87]. Although tumour regression was obtained and thus the surgery avoided, this indication was based on limited observations, mainly case reports, and future investigations are necessary to ponder the risk of infections and malignant tumours associated with this therapy [88]. In any case, the proper therapy should be discussed individually by an interdisciplinary medical team.

Regarding the evolution of arrhythmias in the persisting tumours, a conservative treatment such as observation or antiarrhythmic medication might be recommended [45]. Additionally, children with intractable arrhythmias might benefit from catheter or surgical ablation, defibrillators or pacemakers [45,89]. The increasing understanding of the mTOR pathway activation in the pathophysiology of TS resulted in an increased use of mTOR inhibitors for the management of the patients with TS, rhabdomyomas and refractory arrhythmias [42,86,90,91].

A retrospective multicenter study on 17 patients has demonstrated that mTOR inhibitor treatment is safe in patients with TS under the age of 2 years and has beneficial effects on cardiac manifestations resulting in rhabdomyoma volume reduction and disappearance of arrhythmic events [92].

## 6. Conclusions

Cardiovascular manifestations in tuberous sclerosis are various and potentially severe. Careful screening for cardiac rhabdomyomas, arrhythmias and vascular aneurysms is required when a patient is diagnosed with tuberous sclerosis. Thus, the referral of children with a suspicion of tuberous sclerosis to a tertiary medical center with an interdisciplinary team, including pediatric cardiologists, is of high importance for the appropriate management of the disease. Close surveillance of the cardiovascular manifestations involves multimodal diagnosis techniques (echocardiography, ECG, Holter ECG monitoring, vascular ultrasound, computed tomography, and magnetic resonance angiography). mTOR inhibitors may represent effective therapeutic options in selected cases, such as malignant arrhythmia or obstructive rhabdomyomas, but further studies are needed for dose standardization and duration of treatment.

## Figures and Tables

**Table 1:** Foetal cardiac rhabdomyoma in tuberous sclerosis.

Study	Fetuses with Single Cardiac Rhabdomyoma		Fetuses with Multiple Cardiac Rhabdomyoma	
	Without TS No.	With TS No. (%)	Without TS No.	With TS No. (%)
Peng et al. [17]	9	3 (33.3)	4	35 (89.7)
Sciacca et al. [21]	0	0 (0)	0	10 (100)
Gu et al. [22]	2	0 (0)	0	13 (100)
Webb et al. [23]	N/A	N/A	0	13 (100)

TS: tuberous sclerosis; N/A: not applicable.

**Table 2:** Incidence, localization, and evolution of cardiac rhabdomyomas in children with TS.

Study	Patients with Cardiac RM/Total No.	Incidence of Cardiac RM (%)	Localization				Regression of Cardiac RM in the First 2 Years (%)
			Atria (%)	IVS (%)	LV (%)	RV (%)	
Pearsson et al. [13]	31/40	77.5	N/A	N/A	N/A	N/A	80
Sciacca et al. [21]	33/33	100	2	39.2	26.5	24.5	31
Webb et al. [23]	10/10	100	13	54	73	73	N/A
Wilbur et al. [27]	28/81	35	N/A	N/A	N/A	N/A	N/A
Al Kindi et al. [28]	5/5	100	20	N/A	60	80	N/A
Mettin et al. [32]	18/20	90	N/A	N/A	N/A	N/A	61.1
Au et al. [37]	114/247	46.2	N/A	N/A	N/A	N/A	N/A
Mühler et al. [38]	14/21	66.7	7.1	N/A	100	7.1	42.1

RM: rhabdomyomas; IVS: interventricular septum; LV: left ventricle; RV: right ventricle; N/A: not applicable.

**Table 3:** Electrocardiographic findings in children with tuberous sclerosis.

Study	Electrocardiographic Findings
**Supraventricular and Ventricular Arrhythmias**	
Jawad et al. [25]	premature atrial contractions
Karatza et al. [39]	paroxysmal supraventricular tachycardia
Castilla Cabanes et al. [40], O’Callaghan et al. [41]	Wolff-Parkinson-White syndrome
Silva-Sanchez et al. [42]	sinoatrial nodal re-entrant tachycardia
Howell et al. [43]	atrial flutter
Jawad et al. [25]	junctional rhythm
Mettin et al. [20], Motoki et al. [44]	premature ventricular contractions
Hinton et al. [45]	ventricular tachycardia
**Conduction Defects**	
Cowley et al. [46]	sinus node dysfunction
Mettin et al. [20]	second- and third-degree atrioventricular block
De Wilde et al. [47]	incomplete and complete right bundle branch block
Wacker-Gussmann et al. [48]	prolonged PR interval
**Other ECG changes**	
Shiono et al. [49]	ventricular hypertrophy
Wacker-Gussmann et al. [48]	nonspecific changes of ST interval
Aslan et al. [50]	dome-shaped T waves
Facin et al. [51]	dome–shaped ST–segment elevation
Nguyen et al. [52]	Brugada phenocopy
Janson et al. [53]	widened QRS

**Table 4:** Incidence of arrhythmia in children and young adults with TS according to the type of electrocardiogram changes.

Characteristic	Pearsson et al. [13]	Sciacca et al. [21]	Webb et al. [23]	Wilbur et al. [27]	Mettin et al. [32]	Muhler et al. [38]
No. of evaluated patients	40	33	15	81	20	20
Patients age range	0–18 yrs	antenatal to 12 mos	antenatal to 10 mos	0.2–23.2 yrs	0–18 yrs	0–16 yrs
Atrial/ventricular ectopic beats, %	N/A	24.2	13.3	N/A	20	20
WPW syndrome, %	14.7	6	20	N/A	10	5
Supraventricular tachycardia, %	5.5	N/A	20	2.4	N/A	N/A
Ventricular tachycardia, %	2.8	N/A	N/A	1.23	N/A	N/A
Bradycardia/AVB, %	N/A	N/A	6.7	N/A	N/A	5
RBBB, %	2.8	N/A	N/A	N/A	N/A	N/A
Repolarization disturbances, %	N/A	N/A	N/A	N/A	N/A	20

Abbreviations: yrs: years; mos: months; WPW: Wolff-Parkinson-White; AVB: atrio—ventricular block; RBBB: right bundle branch block; N/A: not applicable.

## Data Availability

Not applicable.
